# 

**Published:** 1901-06-15

**Authors:** 


### ANNOUNCEMENTS.

### NATIONAL DENTAL ASSOCIATION.

The fourth annual meeting of the National Dental Association will be held in Milwaukee, Wis., commencing Tuesday, August 6th, continuing four days. The Masonic Temple Hall, which is conveniently located and especially suited to the various needs of the Association, has been secured.

Special railroad rates are being secured and will be announced later. All regularly organized dental associations are entitled to one delegate for each ten members, and these associations are urged to send full delegations.

Dr. G. V. I. Brown, of Milwaukee, chairman of local committee, will engage rooms at the hotels and answer questions regarding local arrangements.

G. V. Black,
*President.*
J. D. Patterson,
*Chairman Ex. Com.*
A. H. Peck,
*Secretary.*

### AMERICAN DENTAL SOCIETY OF EUROPE.

At the Easter meeting of the American Dental Society of Europe, held at Cologne, the following officers were elected: President, Dr. W. E. Royce, Tunbridge Wells; Vice-President, Dr. F. Foerster, Berlin; Hon. Treasurer, Dr. Wm. A. Spring, Dresden; Hon. Secretary, Dr. L. J. Mitchell, London.

The next meeting is to be held in Stockholm during August, 1902.

L. J. Mitchell,
*Hon. Secretary.*

### OFFICERS OF ILLINOIS STATE DENTAL SOCIETY.

President, M. L. Hanaford; Vice-President, J. E. Hinckins; Secretary, A. H. Peck, 92 State St., Chicago; Treasurer, C. N. Johnson; Executive Council, J. R. Rayburn, W. E. Holland, J. G. Reid; Executive Committee, J. W. Cormany; Board of Examiners, Edmond Noyes. Next meeting will be held at Springfield.

### OFFICERS OF DETROIT DENTAL SOCIETY.

President, M. E. Stafford; Vice-President, J. S. Hall; Secretary, L. N. Hogarth; Treasurer, C. P. Wood.

